# A specific microbiota signature is associated to various degrees of ulcerative colitis as assessed by a machine learning approach

**DOI:** 10.1080/19490976.2022.2028366

**Published:** 2022-02-06

**Authors:** Brigida Barberio, Sonia Facchin, Ilaria Patuzzi, Alexander C. Ford, Davide Massimi, Giorgio Valle, Eleonora Sattin, Barbara Simionati, Elena Bertazzo, Fabiana Zingone, Edoardo Vincenzo Savarino

**Affiliations:** a Division of Gastroenterology, Department of Surgery, Oncological and Gastroenterological Sciences, University of Padua, Padua, Italy; b Research & Development Division, University of Padova, Padova, Italy; c Leeds Gastroenterology Institute, St. James’s University Hospital, Leeds, UK; d Leeds Institute of Biomedical and Clinical Sciences, University of Leeds, Leeds, UK; e Department of Biology and Cribi Biotechnology Centre, University of Padova, Padova, Italy; f BMR Genomic, Via Redipuglia 22, Padova, Italy

**Keywords:** Inflammatory bowel disease, microbiota, ulcerative colitis, machine learning

## Abstract

Ulcerative colitis (UC) is a complex immune-mediated disease in which the gut microbiota plays a central role, and may determine prognosis and disease progression. We aimed to assess whether a specific microbiota profile, as measured by a machine learning approach, can be associated with disease severity in patients with UC. In this prospective pilot study, consecutive patients with active or inactive UC and healthy controls (HCs) were enrolled. Stool samples were collected for fecal microbiota assessment analysis by 16S rRNA gene sequencing approach. A machine learning approach was used to predict the groups’ separation. Thirty-six HCs and forty-six patients with UC (20 active and 26 inactive) were enrolled. Alpha diversity was significantly different between the three groups (Shannon index: p-values: active UC vs HCs = 0.0005; active UC vs inactive UC = 0.0273; HCs vs inactive UC = 0.0260). In particular, patients with active UC showed the lowest values, followed by patients with inactive UC, and HCs. At species level, we found high levels of *Bifidobacterium adolescentis* and *Haemophilus parainfluenzae* in inactive UC and active UC, respectively. A specific microbiota profile was found for each group and was confirmed with sparse partial least squares discriminant analysis, a machine learning-supervised approach. The latter allowed us to observe a perfect class prediction and group separation using the complete information (full Operational Taxonomic Unit table), with a minimal loss in performance when using only 5% of features. A machine learning approach to 16S rRNA data identifies a bacterial signature characterizing different degrees of disease activity in UC. Follow-up studies will clarify whether such microbiota profiling are useful for diagnosis and management.

## Introduction

Ulcerative colitis (UC) is a chronic disorder characterized by inflammation of the gastrointestinal tract, with relapsing and remitting phases, and is associated with a reduced quality of life.^1–3^ The pathogenesis is partially understood, but it has been hypothesized that it arises from dysregulation of the innate and adaptive immune systems,^4^ leading to an abnormal inflammatory response to commensal bacteria in a genetically susceptible individual.^5^ Therefore, a perturbation of the structure of the gut microbiota seems to play a key role in determining intestinal inflammation.

Recent investigations based on 16S rRNA gene sequencing showed significant differences between the microbiota of patients with inflammatory bowel disease (IBD) and healthy controls, suggesting a potential role of gut microbiota not only in the development, but also in determining prognosis and disease progression.^6^ In particular, the dysbiosis associated with UC is characterized by reduced bacterial diversity, a decline in Firmicutes such as *Faecalibacterium prausnitzii* and other short chain fatty acid (SCFA)-producing bacteria, and an increase in Proteobacteria.^7–9^ Despite technological advancements in microbiota analysis, such as next-generation sequencing, high-throughput omics data generation, and molecular networks opening up new horizons in microbial research, the complex relationship between the gut microbiota and IBD is poorly understood.^10^ Indeed, although a potential role of gut microbiota dysbiosis has been widely recognized in IBD, causality is yet to be established. In addition, data on the composition of the gut microbiota in patients with IBD vary widely among studies.^11–13^ Thus, most have failed to observe a specific microbiota signature in association with either IBD type or severity.^14^

Recently, machine learning models have grown in popularity among microbiome researchers because they can effectively account for the interpersonal microbiome variations and the ecology of disease.^15^ However, data in the field of IBD remain limited. A machine learning approach could assist in both the diagnosis and prediction of disease course of patients with IBD. Also, it could also be used to predict response to therapy and drug-related adverse effects.^16^ In addition, machine learning could be applied to profile stool samples revealing specific microbiota signatures to further study a disease specific causal link.^16^

Therefore, we decided to perform a pilot, monocentric, prospective study to assess whether a specific microbiota profile was associated with disease severity in patients with UC. Thus, also with the application of the available machine learning approaches, we investigated how the microbiota profile could be used as a noninvasive marker for monitoring and predicting outcomes in IBD.

## Methods

### Study populations, sample and data collection

We recruited consecutive patients with a histologically confirmed diagnosis of UC for at least 6 months, both with inactive and active disease, from the IBD Unit of Padova Hospital (Italy) from April 2019 to February 2020. Moreover, data about an historical control group of healthy controls (HCs) was used for analysis comparison.^9^ Inclusion and exclusion criteria are reported in Box 1.

**Box 1.** Inclusion and exclusion criteria

Healthy Controls Inclusion Criteria: The Healthy controls enrolled were subjects of both sexes, aged ≥18 years, of Italian nationality, with no relatives with UC, and who did not present evidence of illness on the basis of the anamnestic data collected.
Patients with Inactive Disease Inclusion Criteria Patients with inactive disease were subjects of both sexes Inactive disease was assessed by clinical evaluation and endoscopy with biopsies Patients with a total Mayo score <3 or partial Mayo score <2, with Mayo endoscopic subscore of 0-1 and inactive histological disease (according to Robarts index), and with fecal calprotectin <250 µg/g.
Patients with Active Disease Inclusion Criteria Patients with active disease were subjects of both sexes Active disease was assessed by clinical evaluation and endoscopy with biopsies Patients with total Mayo score >10 or partial Mayo score >7, with Mayo endoscopic subscore of 3 and active histological disease (according to Robarts index), and with fecal calprotectin ≥250 µg/g.
UC Patients (Both Active and Inactive) Exclusion Criteria Under 18 years of age Pregnancy Prior proctocolectomy Presence of stoma Concomitant treatment with antibiotics, prebiotics, steroids, biological therapies, thiopurines or methotrexate, or anticoagulant drugs. Only treatment with mesalazine was allowed.

For each patient, a stool sample was collected for fecal microbiota assessment analysis and for fecal calprotectin (FC) analysis. For patients with inactive UC, fecal samples were collected at home on the evening before or the morning of each visit and stored at 4°C. Upon arrival at the hospital, samples were frozen at −80°C, in all cases within 24 hours of defecation, for the analysis of microbiota. Similarly, fecal samples of HCs were collected at home on the evening before, or the morning of, its delivery to our laboratory, again within 24 hours of defecation. For patients with UC with moderately-to–severely active disease, fecal samples were collected at home if they were not hospitalized, or were collected within 24 hours of hospitalization in the case of hospitalized patients.

The following data were recorded for each patient with UC at baseline: age, gender, age at diagnosis, disease duration, disease location and extent, previous biological treatments, and presence of extraintestinal manifestations. Clinical activity was measured using a total and partial Mayo (p-Mayo) score, while the Mayo endoscopic subscore was used to assess endoscopic activity. For the purpose of the study, all endoscopic examinations were performed within 3 to 5 days of stool collection.

### Sample processing and sequencing

For the microbiota analysis, the stool samples were solubilized and stabilized by degradation in Xpedition Buffer (Zymo Research) and stored at −80°C until analysis. Sequencing protocol was performed at BMR Genomics srl. Briefly: V3–V4 regions of 16S rRNA gene were amplified using the primers Pro341F: 5′-CCTACGGGNBGCASCAG-3′ and Pro805R: Rev 5′-GACTACNVGGGTATCTAATCC-3′.^17^ Primers were modified with forward overhang: 5′-TCGTCGGCAGC GTCAGATGTGTATAAGAGACAG [locus-specific sequence]-3′ and with reverse overhang: 5′-GTCTCGTGGGCTCGGAGATGTGTA TAAGAGACAG [locus-specific sequence]-3′ necessary for dual-index library preparation, following Illumina protocol *https://web.uri.edu/gsc/files/16s-metagenomic-library-prep-guide-15044223-b.pdf*. Samples were normalized, pooled, and run on Illumina MiSeq with 2 × 300 bp approach.

Fecal calprotectin levels were determined using the ELISA Buhlmann fCAL Turbo (Buhlmann Laboratories AG, Schonenbuch, Switzerland), known to perform with high sensitivity and specificity.^18,19^

### Bioinformatic analysis and statistics

The raw reads underwent a filtering procedure performed within QIIME2 analysis framework (version 2020.2).^20^ The primer removal was done via *cutadapt* plugin, while the quality filtering, denoising and chimera checking steps were performed using DADA2 plugin. Alpha diversity was evaluated on rarefied counts (Richness, Shannon, and Pielou indices; rarefaction level: 28,366), while beta diversity was calculated on normalized counts (Bray-Curtis, Jaccard, Canberra, Weighted and Unweighted Unifrac; counts normalized with GMPR).^21^ The diversity analysis was conducted in *R* (version 3.6.3) using *DiversitySeq* package, and the statistical tests (Kruskal-Wallis) on differences in alpha diversity indices distributions between groups were performed using base *R* functions.

A permutational analysis of variance (PERMANOVA) test on Bay-Curtis dissimilarity was used to test for differences in the microbiota composition between disease status groups (*vegan* package). The ANCOM2 package was then used to perform differential abundance analysis at all taxonomic levels (a conservative detection threshold for differentially abundant taxa of 0.8 was chosen).^22^ Supervised and unsupervised machine learning algorithms were applied to normalized data to explore the possibility of grouping and classifying samples, and to identify the most important taxa for class discrimination. To this aim, we performed the following analyses: hierarchical clustering using Ward algorithm on Canberra distance, non-metric multidimensional scaling (NMDS) on Bray-Curtis distance, random forests (training set: 62 patients; test set: 20 patients) and sparse partial least squares discriminant analysis (sPLS-DA).

Machine learning approaches were also run in *R*, using *stats, phyloseq*,^23^ *randomForest*^24^ and *mixOmics*^25^ packages. With the SPLS-DA, a supervised machine learning approach, it is possible to discriminate Amplicon Sequence Variants (ASVs) that best characterize each group. sPLS-DA analysis identified a subset of discriminant ASVs: for each ASV, a loading value that represents the discriminant power of that ASV in explaining differences among the three different examined conditions (active UC, inactive UC, and HCs) was obtained.

Using a one-way analysis of variance (ANOVA) test we tested data for a possible association between disease status and FC values in patients to see whether FC levels could be considered as an identifier for disease groups and their associated microbiota. A *post-hoc* test (Tukey) was then performed to attribute the observed difference to sub-comparisons between disease statuses.

Finally, we compared demographic, clinical, and biochemical data between inactive and active UC using SPSS for Windows (version 24.0 SPSS Inc., Chicago, IL, USA). In particular, continuous variables were reported as medians with ranges, and categorical variables as frequencies and percentages. Comparison between the two groups was carried out using Mann-Whitney tests for numerical data and χ2 test for categorical data. A p-value ≤0.05 was considered statistically significant.

### Ethical statement

The study was approved by University of Padova’s Ethics Committee as part of a larger study aimed to evaluate disease course and characteristics of patients with IBD from the introduction of biologics in clinical practice (N.3312/AO/14). Written informed consent was obtained from all eligible participants, or their legal representatives, before participation.

## Results

Stool samples were collected from 46 patients with UC (20 active UC and 26 inactive UC) and 36 HCs at IBD Unit of Padua University (Italy). Detailed demographic and clinical characteristics of patients with UC are reported in Table 1. Included HCs had a median age of 37 years, with a male to female ratio of 1:1.

**Table 1.** Characteristics of study population

	Patients with active UC (N = 20)	Patients with inactive UC (N = 26)	P value *
Male, n (%)	14 (70.0)	13 (50.0)	0.21
Age (median and range)	40 (20–77)	56.5 (28–75)	0.01
Age at diagnosis (median and range)	26 (17–71)	38 (13–62)	0.01
Smoker, n (%)	4 (20.0)	6 (23.1)	0.90
BMI (median and range)	23.6 (15.8–27.8)	23.7 (16.3–39.2)	0.65
Disease localization -Proctitis or proctosigmoiditis -Left-sided colitis -Extensive colitis	1 (5.0) 7 (35.0) 12 (60.0)	2 (7.7) 12 (46.1) 12 (46.1)	0.64
Fecal calprotectin µg/g (median and range)	1456.5 (204–3800)	60.0 (2–744)	<0.001
Disease activity (p-Mayo), n (%) -Remission -Mild -Moderate -Severe	--1 (5.0) 19 (95.0)	22 (84.6) 4 (15.4)--	<0.001
Endoscopic Mayo -Remission -Mild -Moderate -Severe	--2 (5.0) 18 (95.0)	24 (92.3) 2 (7.7)--	<0.001
Previous abdominal surgery, n (%)	1 (5.0)	2 (7.7)	0.74
Previous steroids, n (%)	17 (85.0)	22 (84.6)	0.92
Naïve to biological drugs, n (%)	5 (25.0)	3 (11.5)	0.21

* We used Mann-Whitney tests for numerical data and χ2 test for categorical data.

### Pre-processing: from reads to Annotated Amplicon Sequence Variant (ASV) table

A total of 6.724.392 reads (mean: 82,442.02; SD: 31,275.40) were obtained from the sequencing procedure. After the filtering, denoising and chimera checking steps, a total of 3.647.949 (mean: 44,831.48; SD: 11,765.93) non-chimeric reads were retained. Details on read loss at each step can be found in Supplementary Table 1. The resulting ASV table collected 3754 ASVs belonging to 14 phyla, 25 classes, 42 orders, 81 families, 189 genera, and 302 species.

### Metataxonomics results

**Figure 1.**
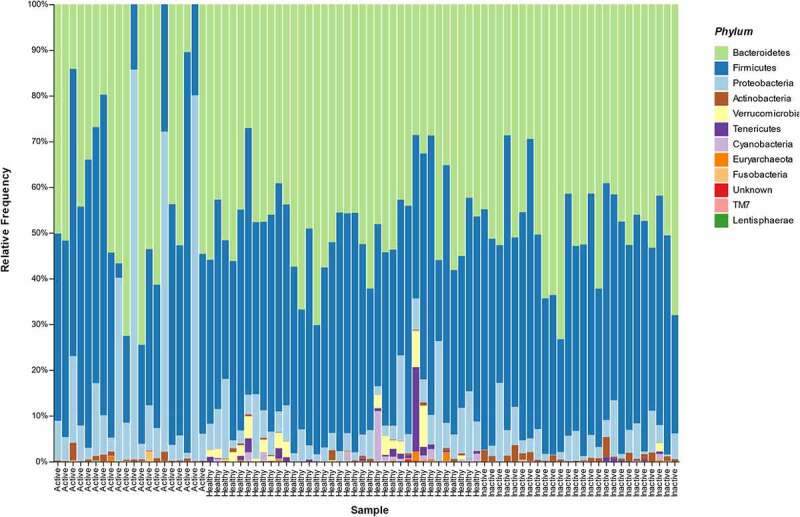
Microbiota composition (Phylum level) in the three different groups of patients (active UC, inactive UC, HCs).

At phylum level, we found that *Tenericutes, Verrucomicrobia, Euryarchaeota* (Archaea) and *Cyanobacteria* characterized the HCs. In particular, *Tenericutes* phylum was increasingly reduced with more active disease, while Verrucomicrobia phylum was absent in active UC. Conversely, *Actinobacteria* was significantly more abundant in patients with both active and inactive UC, and higher levels of *Proteobacteria* were detected consistently in a subset of patients with active UC. Interestingly, in the active UC group there were five patients with a dissimilar microbiota profile compared with the other patients in the same group; demonstrating very high levels of *Proteobacteria* (Figure 1). A more detailed species barplot is provided as Supplementary Figure 1.

At class, order, genus, and species levels we observed differences in microbiota composition between the three groups, summarized in Table 2.

**Table 2.** Bacteria at phylum, class, order, genus, and species level increased and decreased in stool samples of healthy controls, and patients with inactive or active UC

	Healthy Controls	Inactive UC	Active UC
Increased	p__Cyanobacteria p__Tenericutes c_Alphaproteobacteria o__Clostridiales g__Butyricimonas s__Akkermansia muciniphila s__Coprococcus eutactus	g__Holdemania s__Eubacterium dolichum s__Blautia producta s__Ruminococcus gnavus s__Bifidobacterium adolescentis	c_Gammaproteobacteria g__Granulicatella s__Haemophilus parainfluenzae s__Streptococcus anginosus s__Clostridium symbiosum
Decreased	g__Blautia g__Dorea s__Clostridium celatum s__Eubacterium dolichum		p__Tenericutes p__Verrucomicrobia g__Lachnospira g__Oscillospira

UC: ulcerative colitis; p_: phylum level; c_: class; o_: order level; g_: genus level; s_: species level.

### Alpha diversity analysis

**Figure 2.**
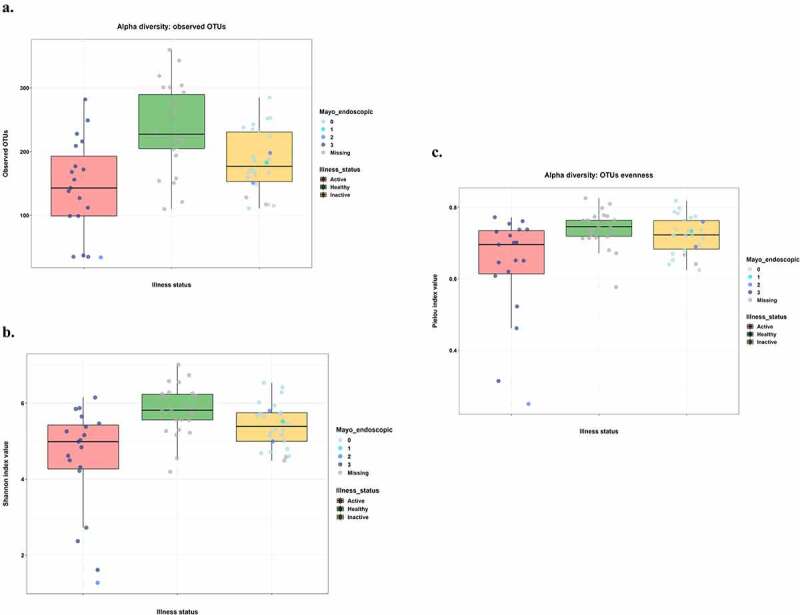
Alpha diversity analysis results for richness (a), Shannon (b), and Pielou (c) indices.

Alpha diversity results (ASV richness, Shannon index, and Pielou index) showed a marked difference among the three groups (Figure 2a, 2b and 2c). In particular, patients with active UC had the lowest values for all the indexes, followed by inactive UC, and HCs. The detected differences were statistically significant for all the pairwise comparisons, both for richness (p-values: active UC vs HCs = 0.0009; active UC vs inactive UC = 0.05; HCs vs inactive UC = 0.008) and for Shannon index (p-values: active UC vs HCs = 0.0005; active UC vs inactive UC = 0.03; HCs vs inactive UC = 0.03). Regarding the Pielou index, the pairwise comparisons were statistically significant between active UC and HCs (p = .004) and between active and inactive UC (p = .05), although the comparison between HCs and patients with inactive UC was not statistically significant (Table 3).

**Table 3.** ASV Richness, Shannon index, Pielou index comparisons

Richness		Shannon index		Pielou index	
Comparison	p-value*	Comparison	p-value*	Comparison	p-value*
Active UC-Healthy	0.0009	Active UC-Healthy	0.0005	Active UC-Healthy	0.004
Active UC-Inactive	0.05	Active UC-Inactive	0.03	Active-Inactive UC	0.05
Healthy–Inactive UC	0.008	Healthy-Inactive UC	0.03	Healthy-Inactive UC	0.14

*Kruskal Wallis pairwise test

Points were colored corresponding to Mayo endoscopic subscores. Thus, we combined each metric quantitative information with endoscopic activity.

Figure 2 and Supplementary Figure 2 show diversity plots for microbiota patient samples (plots of Richness, Shannon index, and Pielou index, respectively) according to disease activity status (based on Mayo Endoscopic score) and fecal calprotectin values, respectively.

After performing the ANOVA test, FC values were statistically significantly different among the three groups (p < .001). *Post-hoc*, with the Tukey test, we demonstrated that there were statistically significant differences between HCs and active UC (p < .001) and between patients with inactive and active UC (p < .001). Conversely, no differences were found between HCs and patients with inactive UC (p = .86).

### Beta diversity analysis

The PERMANOVA analysis based on Bray-Curtis dissimilarity showed that the composition of the microbiota at the ASV level was statistically significantly different among the three groups (p < .001). A hierarchical clustering using Ward algorithm on Canberra distance was performed (Supplementary Figure 3). As shown in the figure, cutting the tree at level 1.15, four different clusters were visible: the first one consisted mainly of HCs; the second consisted of five patients with active UC; the third mainly of patients with inactive UC; and the final cluster consisting of stool samples belonging to all groups (active and inactive UC and HCs) demonstrating similar microbial composition.

### Non-metric multidimensional scaling

**Figure 3.**
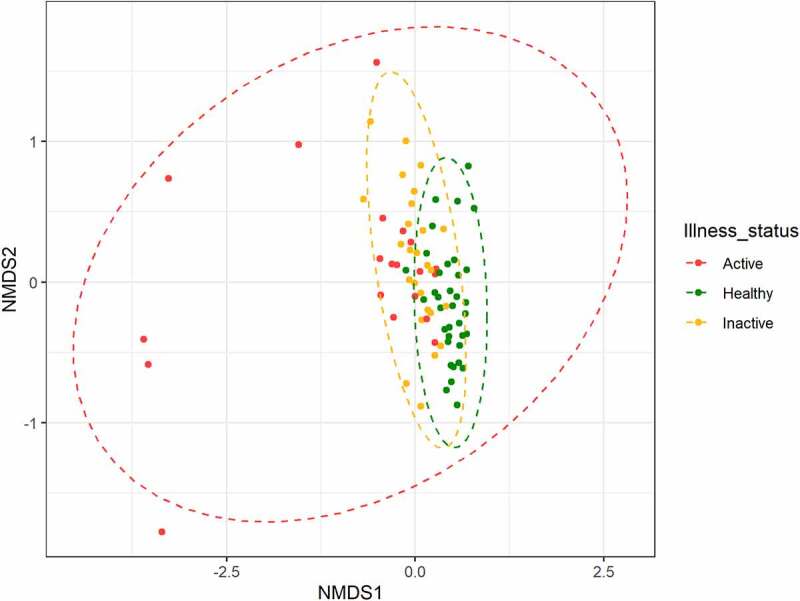
Non metric multidimensional scaling (NMDS) plot of Beta diversity (Bray-Curtis distance matrix).

After the ASV table construction, an ordination graphical analysis was performed to represent the highest possible fraction of the complete information into a bidimensional plot (NMDS) based on the Bray-Curtis distance between samples (Figure 3). Beta-diversity based on Bray-Curtis distances showed a marked disease-associated pattern, with samples from patients with active UC located furthest from HCs and patients with inactive UC. These last two groups, again, were slightly separated from each other in the graph. Interestingly, the aforementioned five patients with active UC, and with a very dissimilar microbiota composition, were placed furthest from other patients in the same group.

Each point represented the gut microbiota of a patient while colors represent each clinical status (healthy, inactive UC, active UC). Patients with similar microbiota composition tended to be in the same area of the graph, while points far apart from each other represent patients with dissimilar microbiota.

### sPLS-DA (Sparse partial least squares discriminant analysis) and random forest analyses

**Figure 4.**
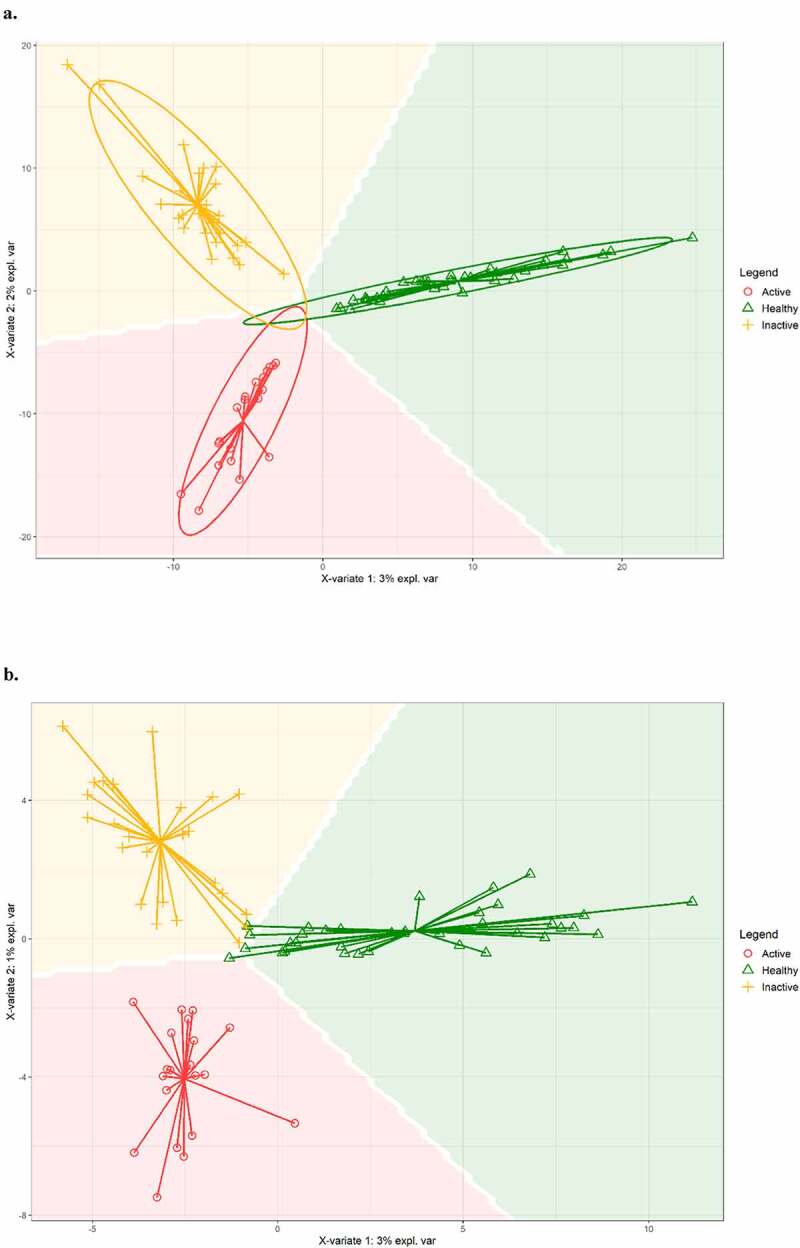
Sparse Partial Least Squares Discriminant Analysis (SPLS-DA), a machine learning-supervised approach using all ASVs (a) and 5% of all ASVs (b).

The sPLS-DA analysis allowed us to observe a perfect class prediction and group separation using the complete information (full ASV table) (Figure 4a), with a minimal loss in performance when using only the 5% of most important features (Figure 4b). Interestingly, the majority of the features selected by the sPLS-DA algorithm were the ones marked as differentially abundant among groups. The host trait predictive potential of the microbiota was also confirmed by a Random Forest analysis (Supplementary Table 2).

## Discussion

Bacterial dysbiosis is one of the most widely proposed etiological factors in IBD,^26^ with variations affecting the α-diversity and abundance of phyla, families and genera.^26^ However, it remains unclear whether alterations in the intestinal microbiota are a cause or an effect of inflammation in IBD, as well as how the microbiota changes based on intestinal inflammation. In our study, we aimed to assess whether a specific microbiota profile, as measured by a machine learning approach, was associated with disease severity in patients with UC. We also aimed to verify if it could be used as a noninvasive marker for IBD monitoring, as well as a predictor of outcome before it become clinically manifested.

Overall, in our study, fecal microbiota alpha diversity (ASV richness, Shannon index, and Pielou index) was significantly reduced in patients with UC compared with HCs, and this difference remained statistically significant between patients with active and inactive disease. The gut microbiota of the HCs, at phylum level, was characterized by high levels of *Tenericutes, Verrucomicrobia, Euryarchaeota*, and *Cyanobacteria*. While, *Actinobacteria* were significantly more abundant in patients with both active and inactive UC and higher levels of *Proteobacteria* were detected in a consistent subset of patients with active UC. Of the microbial species found to be significantly increased in HCs, there were *Akkermansia muchiniphila* and *Coprococcus eutactus*, which were practically absent in active UC. On the other hand, among the microbial species found to be significantly higher in active UC, there were *Haemophilus parainfluenzae, Clostridium symbiosum, Clostridium perfringens*, and *Streptococcus anginosus*. In addition, at genus level, we found high levels of *Granulicatella*. Regarding inactive UC, we observed higher levels of *Ruminococcus gnavus, Blautia producta, Eubacterium dolichum*, and *Bifidobacterium adolescentis*. Moreover, at genus level, in inactive UC *Holdemania* was significantly increased. The application of sPLS-DA, a machine learning-supervised approach, allowed us to observe a perfect class prediction and group separation using the complete information, with a minimal loss in performance when using only 5% of features. Overall, machine learning approaches highlighted a disease-state signature that agreed well with differential abundance analysis results.

Numerous studies have supported evidence for intestinal dysbiosis in IBD patients compared with healthy controls,^11,12^ and our results confirmed this finding. As our results also show, current literature reports that the gut microbiota of healthy individuals is dominated at phylum level by the bacterial phyla Firmicutes and Bacteroidetes, and to a lesser extent by Proteobacteria, Actinobacteria, and Verrucomicrobia.^13^ In fact, a relevant abundance of Akkermansia muchiniphila, belonging to Verrucomicrobia phylum, was found in our controls, whereas it was very low in our patients with UC, especially those with active disease. Interestingly, we did not find a statistically significant difference in *Faecalibacterium prausnitzii* in patients with UC compared with healthy controls, or between active and inactive UC, in contrast with current literature.^27^ Fecal samples of patients with active UC had a lower abundance of *F. prausnitzii*, but this difference was not statistically significant.

Several studies using meta-genomics analysis have demonstrated that members of the phylum Firmicutes are less abundant in patients with UC or CD.^11,28,29^ Among *Firmicutes, Clostridium clusters XIVa and IV* are largely underrepresented in the gut of patients with IBD. *Clostridium cluster XIVa* comprises species belonging to the *Clostridium, Ruminococcus, Lachnospira, Roseburia, Eubacterium, Coprococcus, Dorea*, and *Butyrivibrio* genera.^30–32^ We found that *g_Ruminococcus* was lower in UC, mostly in those with active disease, compared with controls. Likewise, *Coprococcus eutactus* was statistically higher in HCs compared with patients with UC. Previous studies have evaluated microbial differences between patients with active and inactive IBD.^27^ A decrease in the Clostridium family was found in active UC compared with inactive UC and healthy controls.^33^ Furthermore, a decrease of *Clostridium coccoides* and *Clostridium leptum* was reported in the feces of patients with active compared with inactive UC.^34^ Interestingly, in patients with active UC we found high levels of *Clostridum perfringens* and *symbiosum* compared with patients with inactive UC and controls. Several microbiome analyses have revealed there is an expansion of the *Proteobacteria phylum* in patients with IBD.^11,28^ In keeping with these findings, we demonstrated a relevant and significant increase of *Proteobacteria* in patients with active UC. In particular, we observed high levels of *Hemophilus parainfluenzae*, a *Gammaproteobacteria*, at class level. It is worth of noting that our samples demonstrated a specific signature even in patients with inactive UC, who demonstrated higher levels of *Ruminococcus gnavus, Blautia producta, Eubacterium dolichum*, and *g*_*Holdemania*, all belonging to *Firmicutes phylum*, and *Bifidobacterium adolescentis*, belonging to *Actinobacteria phylum*. Another recent study by Clooney *et al*. applied a machine learning approach to stool samples of patients with both active and inactive IBD. They recruited 303 patients with CD, 228 with UC, and 161 controls, and demonstrated that machine learning separated IBD from controls, and active from inactive IBD, when consecutive time points were modeled.^16^

Our study had some limitations. First, the sample size is relatively small. However, this drawback is balanced by the fact that we enrolled a study population as homogeneous as possible. In particular, we tried to limit the influence of ongoing treatments on microbiota characterization and we divided our patients based on their disease activity. Indeed, we included patients who were taking only mesalazine, and not biological drugs or immunosuppressants. In addition, although our cohort is homogeneous because it belongs to a single center, a validation cohort from multiple sites will be required in future studies to determine the quality of the machine learning. However, we are aware that also dietary factors, which we did not consider, could lead to microbiota variability in patients with IBD and controls. Another limitation is the cross-sectional design of the study, with a lack of longitudinal data to further support our findings. Moreover, we only assessed the fecal microbiota profile, without examining the microbiota adherent to the colonic mucosa. Finally, we did not match the microbiota of both active and inactive patients based on characteristics such as diet or smoking or alcohol consumption. On the other hand, we considered as exclusion criteria the medications (antibiotics and probiotics) known to influence the microbiota assessment in such patients.

However, despite these limitations, the distinct signatures observed for the gut microbiota in active and inactive UC could have several implications. Firstly, these results further strengthen the association between dysbiosis and IBD and have given a proof-of-concept for a potential correlation between disease severity and degree of dysbiosis. Secondly, since the accuracy of diagnosis in IBD is key to commencing prompt and effective treatment, there is an urgent need to develop a novel classification technique that can expedite IBD diagnosis, before intestinal damage ensues. In our study, we used supervised and unsupervised machine learning algorithms on an IBD associated metagenomics data, which might improve diagnostic accuracy and elucidate which subsets of microbiota are most informative to identify these patients. Potentially, all of this could enable the identification of individuals who will develop IBD before symptoms, using their microbiota. Finally, finding a specific microbiota signature will represent an opportunity for personalized prognostics or therapeutics based on microbiota manipulation.

In conclusion, in our study, the application of sPLS-DA, a machine learning-supervised approach, allowed us to observe a perfect class prediction and group separation using the complete information (full OTU table), with a minimal loss in performance when using only 5% of features. In addition, the majority of the features selected by the sPLS-DA algorithm were the ones marked as differentially abundant between groups. Our data support the concept that implementing current 16S rRNA data will be helpful to improve management of IBD patients. Further follow-up studies will aim to clarify whether such microbiota profiling also predicts disease outcomes.

### List of abbreviations

ASVs: Amplicon Sequence VariantsCD: Crohn’s diseaseHBI: Harvey-Bradshaw indexHCs: Healthy controlsIBD: inflammatory bowel diseaseFC: fecal calprotectinNMDS: non-metric multidimensional scalingp-MAYO: partial Mayo ScoresPLS-DA: sparse partial least squares discriminant analysisUC: ulcerative colitis

## Supplementary Material

Supplemental Material

## Data Availability

The data underlying this study is available within the manuscript and supplementary materials
