# Supplementary material for: A specific microbiota signature is associated to various degrees of ulcerative colitis as assessed by a machine learning approach
Source: Gut Microbes. 2022 Feb 6;14(1):2028366. doi: 10.1080/19490976.2022.2028366 (PMC8820804; doi:10.1080/19490976.2022.2028366)
Supplement: Supplemental Material [file KGMI_A_2028366_SM1844.zip › supplementary/Supplementary Materials_Gut microbes.docx]

**Supplementary Table 1. Details on read passing each DADA2 pre-processing step. Samples are ordered according decreasing number of final non-chimeric reads.**

| **sample** | **input** | **filtered** | **% input passed filter** | **denoised** | **merged** | **% input merged** | **non-chimeric** | **% input non-chimeric** | **Group** |
| --- | --- | --- | --- | --- | --- | --- | --- | --- | --- |
| s42 | 94438 | 77312 | 81.87 | 77089 | 76252 | 80.74 | 69988 | 74.11 | Active |
| s68 | 105742 | 82200 | 77.74 | 80881 | 78338 | 74.08 | 66894 | 63.26 | Healthy |
| s12 | 81040 | 71372 | 88.07 | 70489 | 69012 | 85.16 | 66657 | 82.25 | Inactive |
| s37 | 92729 | 78376 | 84.52 | 77720 | 76595 | 82.6 | 64936 | 70.03 | Active |
| s33 | 91787 | 75803 | 82.59 | 73911 | 71867 | 78.3 | 64616 | 70.4 | Inactive |
| s13 | 80555 | 71599 | 88.88 | 70334 | 68274 | 84.75 | 62479 | 77.56 | Inactive |
| s64 | 214670 | 106041 | 49.4 | 102215 | 92531 | 43.1 | 61707 | 28.75 | Healthy |
| s77 | 135771 | 114880 | 84.61 | 111360 | 102593 | 75.56 | 60889 | 44.85 | Healthy |
| s36 | 83996 | 69184 | 82.37 | 68979 | 68614 | 81.69 | 60810 | 72.4 | Active |
| s26 | 94901 | 84365 | 88.9 | 82972 | 78809 | 83.04 | 60589 | 63.84 | Active |
| s38 | 72831 | 58778 | 80.7 | 58688 | 58599 | 80.46 | 58593 | 80.45 | Active |
| s28 | 70919 | 63122 | 89.01 | 61970 | 60575 | 85.41 | 58500 | 82.49 | Active |
| s27 | 88982 | 79336 | 89.16 | 77620 | 73430 | 82.52 | 57490 | 64.61 | Active |
| s15 | 68953 | 61284 | 88.88 | 60302 | 59092 | 85.7 | 57045 | 82.73 | Inactive |
| s9 | 78231 | 69533 | 88.88 | 68354 | 66323 | 84.78 | 56491 | 72.21 | Inactive |
| s21 | 81575 | 73753 | 90.41 | 72362 | 70009 | 85.82 | 54959 | 67.37 | Inactive |
| s16 | 69271 | 61741 | 89.13 | 60265 | 58687 | 84.72 | 54887 | 79.24 | Inactive |
| s76 | 103700 | 86091 | 83.02 | 79177 | 71071 | 68.54 | 53712 | 51.8 | Healthy |
| s32 | 93957 | 77489 | 82.47 | 74136 | 69317 | 73.78 | 52439 | 55.81 | Active |
| s20 | 76771 | 66615 | 86.77 | 65578 | 64146 | 83.55 | 52424 | 68.29 | Inactive |
| s18 | 83276 | 73265 | 87.98 | 72212 | 69882 | 83.92 | 52409 | 62.93 | Active |
| s43 | 69446 | 58362 | 84.04 | 56931 | 54932 | 79.1 | 51947 | 74.8 | Active |
| s57 | 97654 | 77247 | 79.1 | 74303 | 68522 | 70.17 | 51768 | 53.01 | Healthy |
| s23 | 74661 | 66652 | 89.27 | 64531 | 61424 | 82.27 | 50574 | 67.74 | Inactive |
| s63 | 166862 | 102645 | 61.51 | 97842 | 86032 | 51.56 | 50508 | 30.27 | Healthy |
| s19 | 76392 | 66569 | 87.14 | 65267 | 62456 | 81.76 | 49670 | 65.02 | Active |
| s74 | 76739 | 60128 | 78.35 | 55957 | 52698 | 68.67 | 48814 | 63.61 | Healthy |
| s29 | 68130 | 60670 | 89.05 | 59303 | 57228 | 84 | 48671 | 71.44 | Inactive |
| s70 | 88101 | 65538 | 74.39 | 62301 | 58896 | 66.85 | 48650 | 55.22 | Healthy |
| s22 | 58972 | 52987 | 89.85 | 52560 | 51967 | 88.12 | 48460 | 82.17 | Inactive |
| s25 | 73244 | 65154 | 88.95 | 62462 | 58236 | 79.51 | 47620 | 65.02 | Active |
| s8 | 78132 | 69913 | 89.48 | 67800 | 63485 | 81.25 | 47460 | 60.74 | Inactive |
| s35 | 88518 | 74364 | 84.01 | 71370 | 66061 | 74.63 | 47172 | 53.29 | Inactive |
| s56 | 96892 | 75055 | 77.46 | 69837 | 63218 | 65.25 | 47163 | 48.68 | Healthy |
| s78 | 91135 | 75251 | 82.57 | 68931 | 61152 | 67.1 | 46942 | 51.51 | Healthy |
| s2 | 71812 | 64258 | 89.48 | 62476 | 58259 | 81.13 | 46412 | 64.63 | Inactive |
| s10 | 71352 | 62227 | 87.21 | 60062 | 57039 | 79.94 | 46089 | 64.59 | Inactive |
| s67 | 85705 | 76481 | 89.24 | 71790 | 64712 | 75.51 | 45770 | 53.4 | Healthy |
| s53 | 97828 | 77537 | 79.26 | 74482 | 67615 | 69.12 | 45696 | 46.71 | Healthy |
| s58 | 103793 | 81760 | 78.77 | 80237 | 75257 | 72.51 | 45661 | 43.99 | Healthy |
| s40 | 90296 | 74941 | 82.99 | 73380 | 69567 | 77.04 | 45598 | 50.5 | Active |
| s30 | 68502 | 60205 | 87.89 | 57531 | 55172 | 80.54 | 45406 | 66.28 | Inactive |
| s11 | 66262 | 59205 | 89.35 | 58077 | 56361 | 85.06 | 45365 | 68.46 | Inactive |
| s73 | 80490 | 58024 | 72.09 | 54652 | 51843 | 64.41 | 45251 | 56.22 | Healthy |
| s44 | 82067 | 68539 | 83.52 | 66381 | 62243 | 75.84 | 44545 | 54.28 | Active |
| s65 | 78553 | 67760 | 86.26 | 64733 | 60282 | 76.74 | 43976 | 55.98 | Healthy |
| s39 | 81857 | 68926 | 84.2 | 67147 | 63851 | 78 | 43096 | 52.65 | Active |
| s7 | 75955 | 67705 | 89.14 | 63514 | 57984 | 76.34 | 42896 | 56.48 | Inactive |
| s55 | 78615 | 60106 | 76.46 | 57404 | 53497 | 68.05 | 42804 | 54.45 | Healthy |
| s41 | 72944 | 59758 | 81.92 | 58339 | 55563 | 76.17 | 42781 | 58.65 | Active |
| s66 | 73114 | 61624 | 84.28 | 59742 | 56019 | 76.62 | 42409 | 58 | Healthy |
| s72 | 91403 | 66068 | 72.28 | 62544 | 57852 | 63.29 | 42325 | 46.31 | Healthy |
| s75 | 73903 | 60514 | 81.88 | 54590 | 49634 | 67.16 | 42313 | 57.25 | Healthy |
| s45 | 75749 | 62307 | 82.25 | 60025 | 55532 | 73.31 | 42187 | 55.69 | Active |
| s51 | 81626 | 64021 | 78.43 | 59245 | 54363 | 66.6 | 42130 | 51.61 | Healthy |
| s17 | 63764 | 57380 | 89.99 | 55687 | 52894 | 82.95 | 41260 | 64.71 | Active |
| s71 | 79502 | 57712 | 72.59 | 53663 | 49608 | 62.4 | 40853 | 51.39 | Healthy |
| s69 | 76515 | 56979 | 74.47 | 54008 | 50831 | 66.43 | 40498 | 52.93 | Healthy |
| s5 | 54727 | 48929 | 89.41 | 48041 | 46403 | 84.79 | 40238 | 73.52 | Inactive |
| s31 | 82769 | 67427 | 81.46 | 63994 | 57079 | 68.96 | 40187 | 48.55 | Inactive |
| s82 | 86629 | 70863 | 81.8 | 66313 | 59935 | 69.19 | 39731 | 45.86 | Healthy |
| s80 | 77952 | 61849 | 79.34 | 58795 | 54562 | 69.99 | 39194 | 50.28 | Healthy |
| s24 | 71688 | 64392 | 89.82 | 62225 | 58267 | 81.28 | 38775 | 54.09 | Inactive |
| s46 | 45118 | 37550 | 83.23 | 37436 | 37327 | 82.73 | 37105 | 82.24 | Active |
| s14 | 66957 | 58896 | 87.96 | 56647 | 52973 | 79.11 | 35854 | 53.55 | Inactive |
| s34 | 73203 | 56739 | 77.51 | 54824 | 50787 | 69.38 | 35574 | 48.6 | Inactive |
| s59 | 76751 | 60490 | 78.81 | 58306 | 53287 | 69.43 | 34663 | 45.16 | Healthy |
| s79 | 62512 | 49089 | 78.53 | 47918 | 45166 | 72.25 | 32970 | 52.74 | Healthy |
| s3 | 53836 | 48458 | 90.01 | 47286 | 44702 | 83.03 | 32909 | 61.13 | Inactive |
| s1 | 50947 | 44904 | 88.14 | 43649 | 41795 | 82.04 | 32637 | 64.06 | Inactive |
| s4 | 50097 | 44678 | 89.18 | 41341 | 37269 | 74.39 | 28366 | 56.62 | Inactive |
| s50 | 62394 | 47375 | 75.93 | 44705 | 40426 | 64.79 | 27460 | 44.01 | Healthy |
| s81 | 56385 | 40917 | 72.57 | 38509 | 35053 | 62.17 | 25579 | 45.36 | Healthy |
| s62 | 208312 | 45567 | 21.87 | 43304 | 36842 | 17.69 | 24871 | 11.94 | Healthy |
| s52 | 49165 | 34997 | 71.18 | 31449 | 28552 | 58.07 | 24360 | 49.55 | Healthy |
| s6 | 41626 | 37484 | 90.05 | 36176 | 33650 | 80.84 | 23701 | 56.94 | Active |
| s61 | 205296 | 37526 | 18.28 | 34513 | 29938 | 14.58 | 23477 | 11.44 | Healthy |
| s60 | 68687 | 48475 | 70.57 | 46064 | 37759 | 54.97 | 22247 | 32.39 | Healthy |
| s47 | 45015 | 33989 | 75.51 | 31294 | 28014 | 62.23 | 18086 | 40.18 | Healthy |
| s49 | 48086 | 27153 | 56.47 | 25730 | 23025 | 47.88 | 17381 | 36.15 | Healthy |
| s48 | 49072 | 26363 | 53.72 | 23684 | 21145 | 43.09 | 16731 | 34.09 | Healthy |
| s54 | 46588 | 30521 | 65.51 | 27060 | 23353 | 50.13 | 16599 | 35.63 | Healthy |

**Supplementary Table 2. Random Forest confusion matrix. The training test was composed by 62 patients evenly chosen among the three groups. Data coming from the remaining 20 patients were used as test set for performance evaluation.**

|  | **Reference** | | |
| --- | --- | --- | --- |
| **Prediction** | **Active** | **Healthy** | **Inactive** |
| **Active** | 5 | 0 | 0 |
| **Healthy** | 0 | 9 | 0 |
| **Inactive** | 0 | 0 | 6 |

**Supplementary Figure 1.** **Microbiota composition (Species level) in the three different groups of patients (active UC, inactive UC, HCs).**


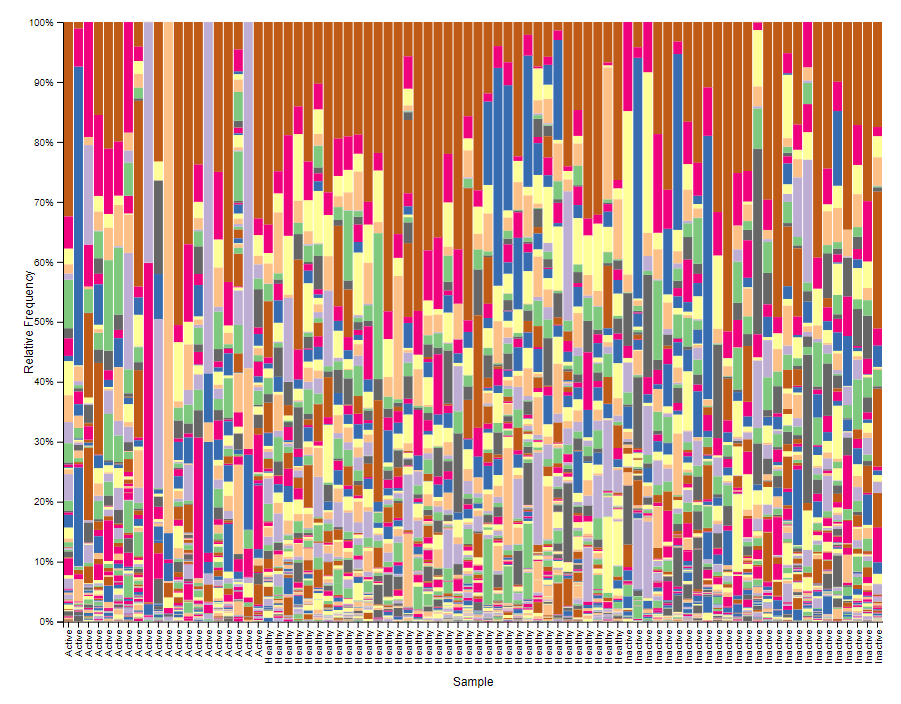


Abbreviations: HCs: healthy controls; UC: ulcerative colitis.


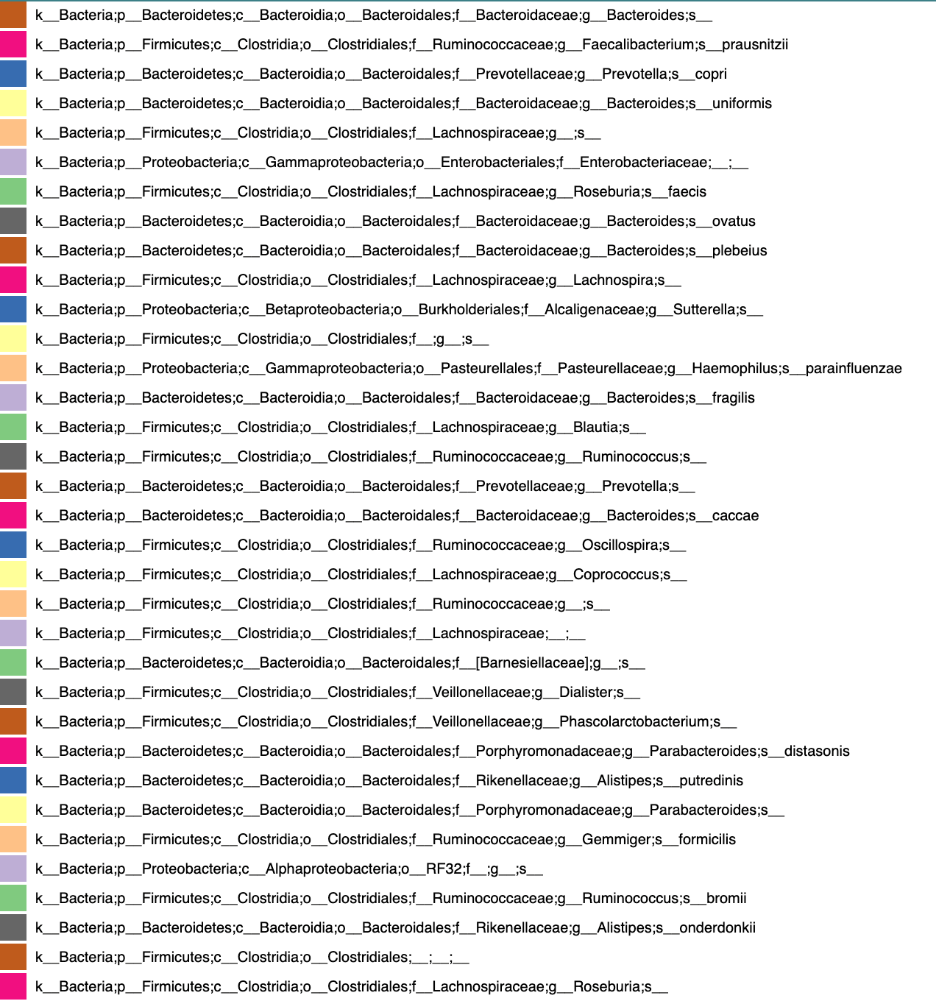


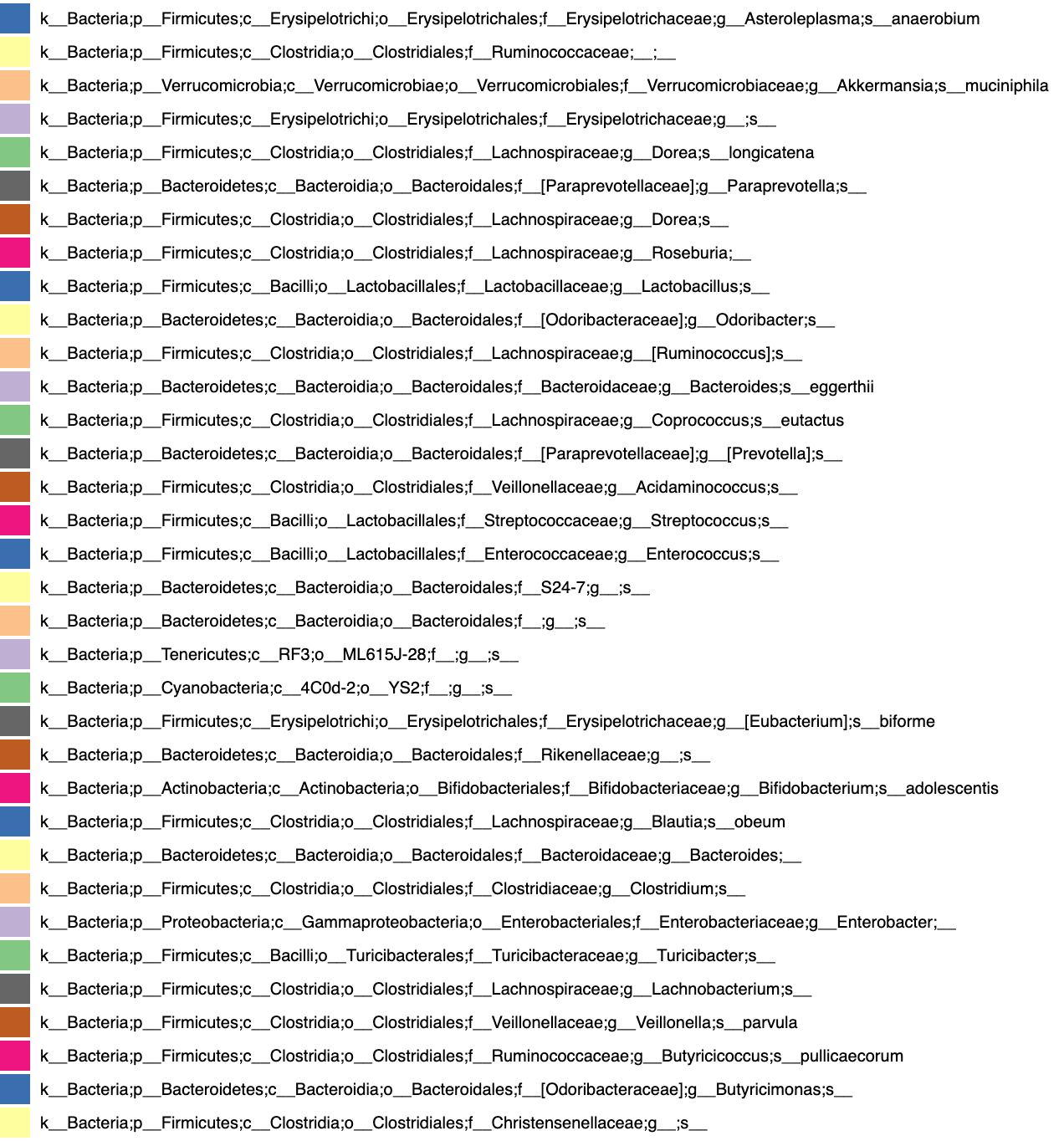


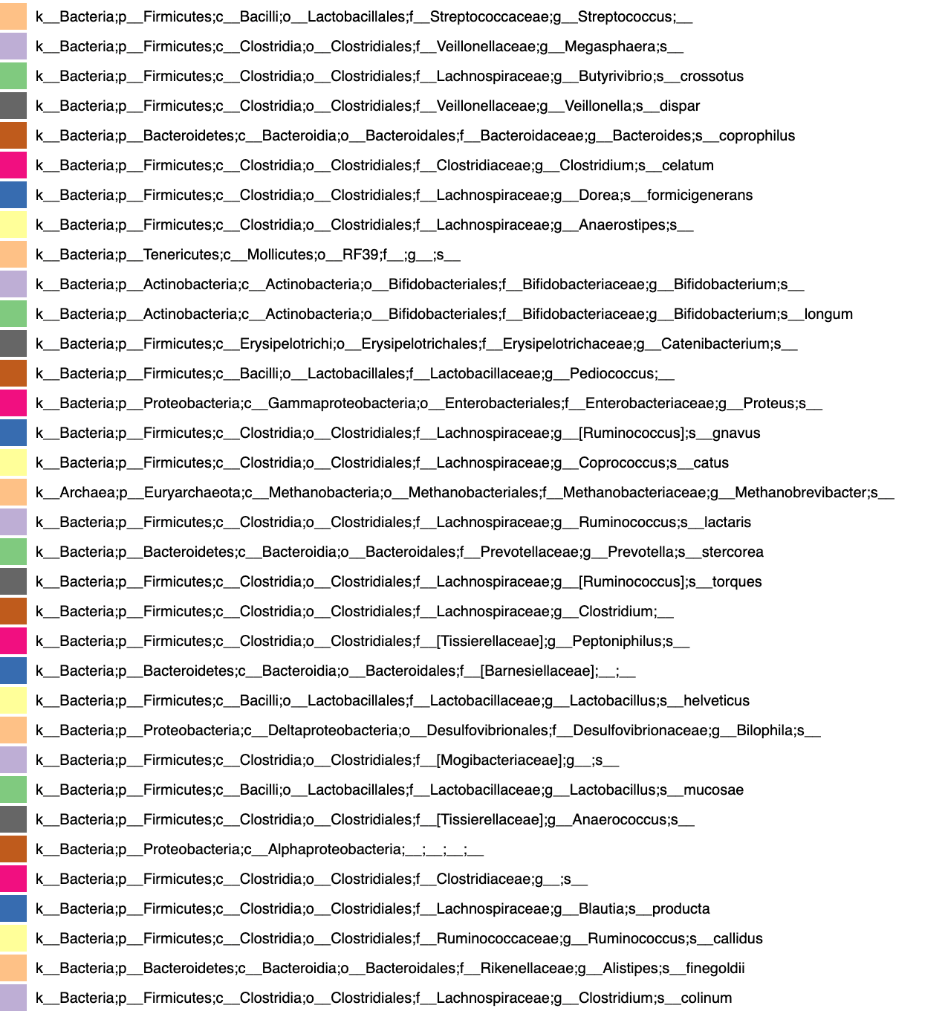


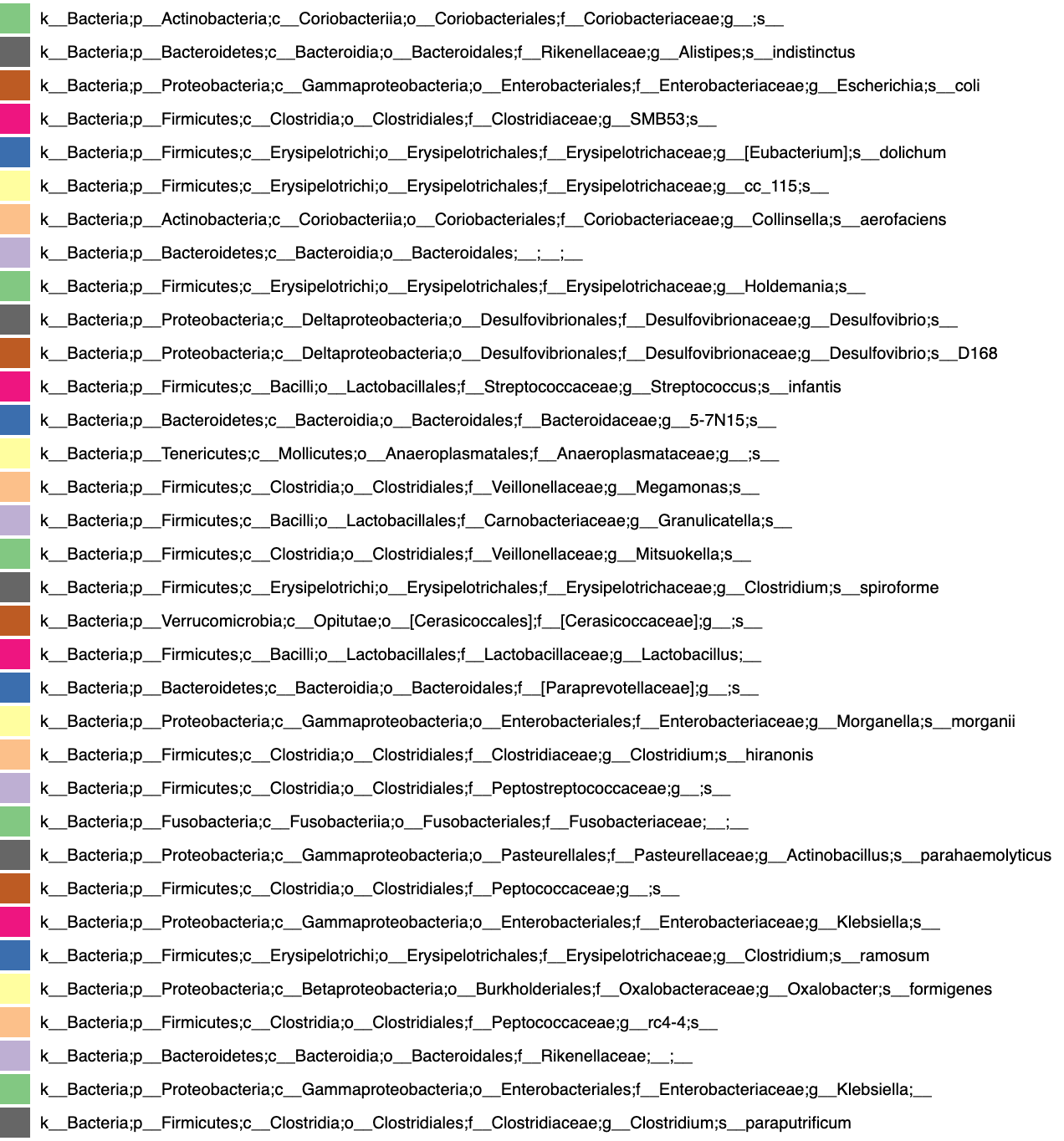


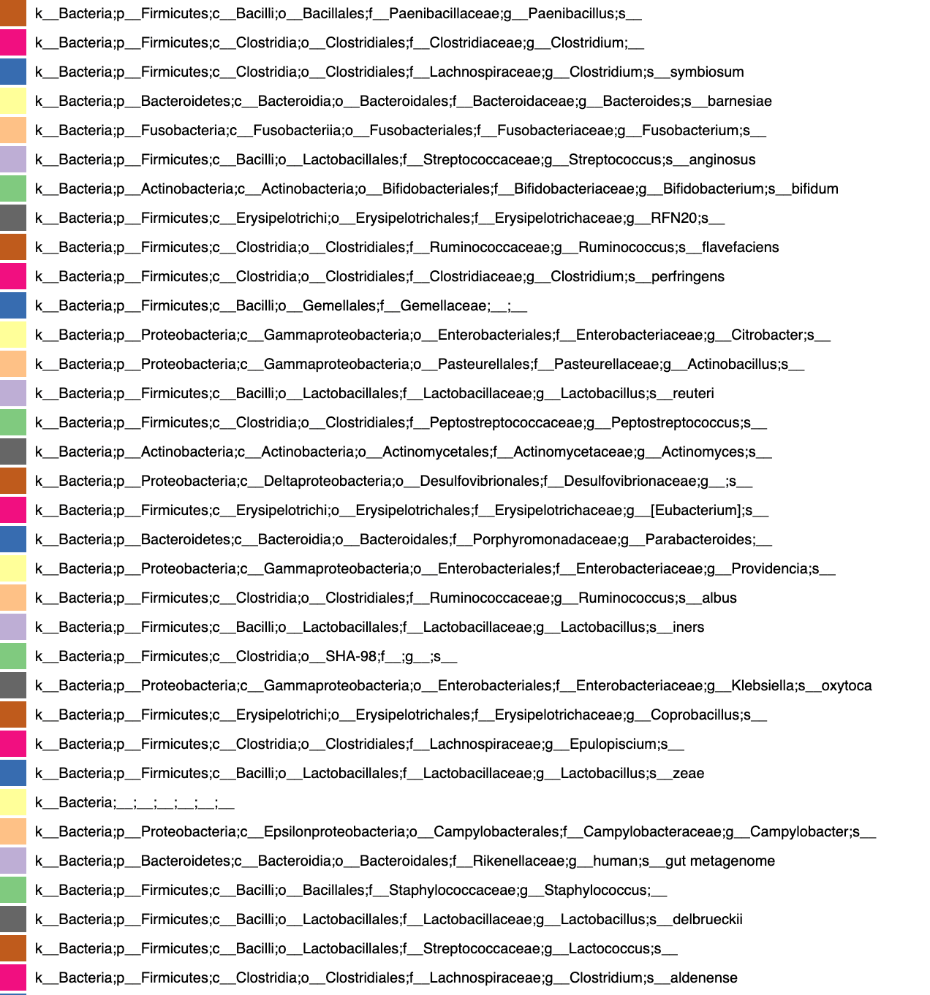


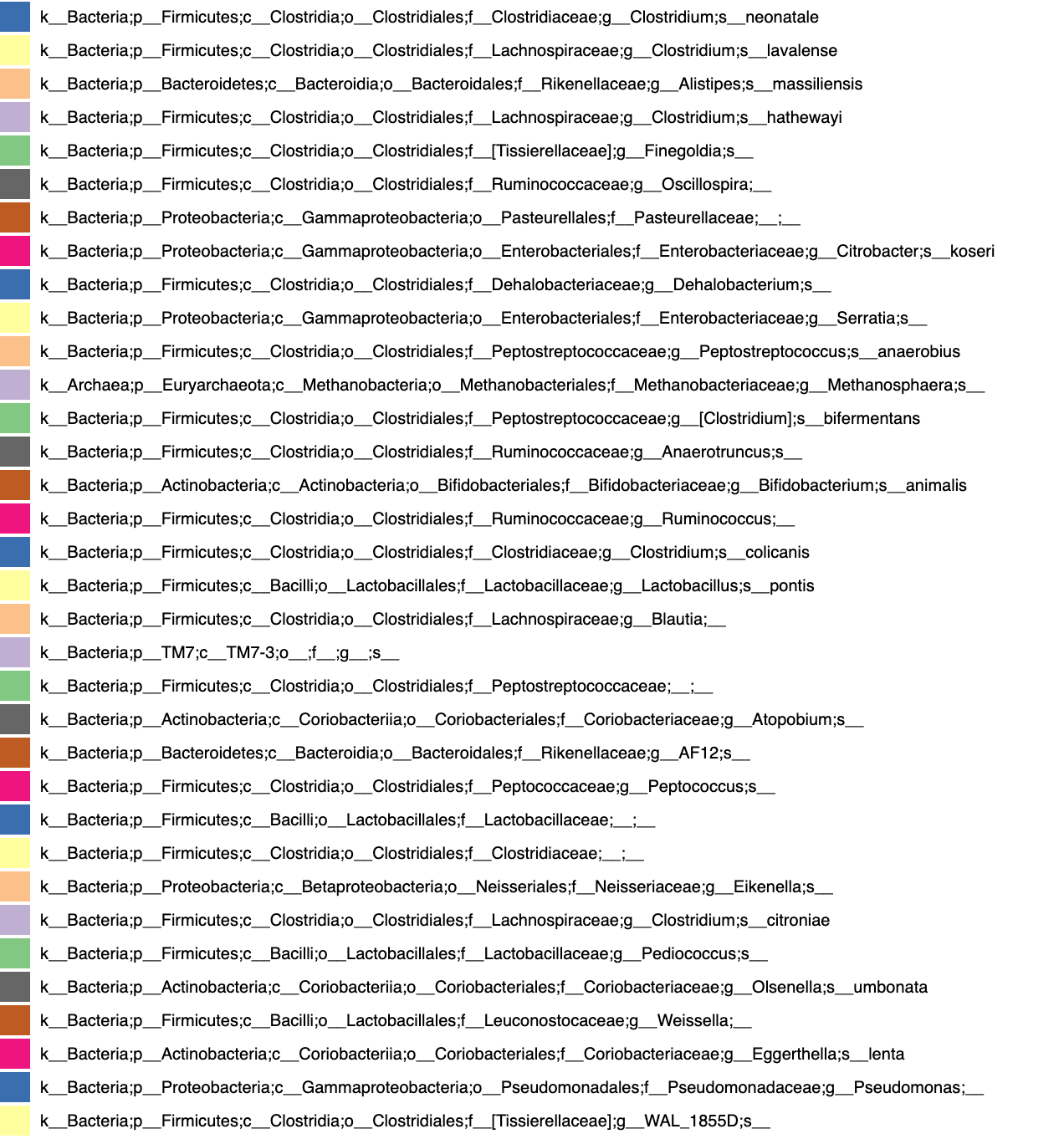


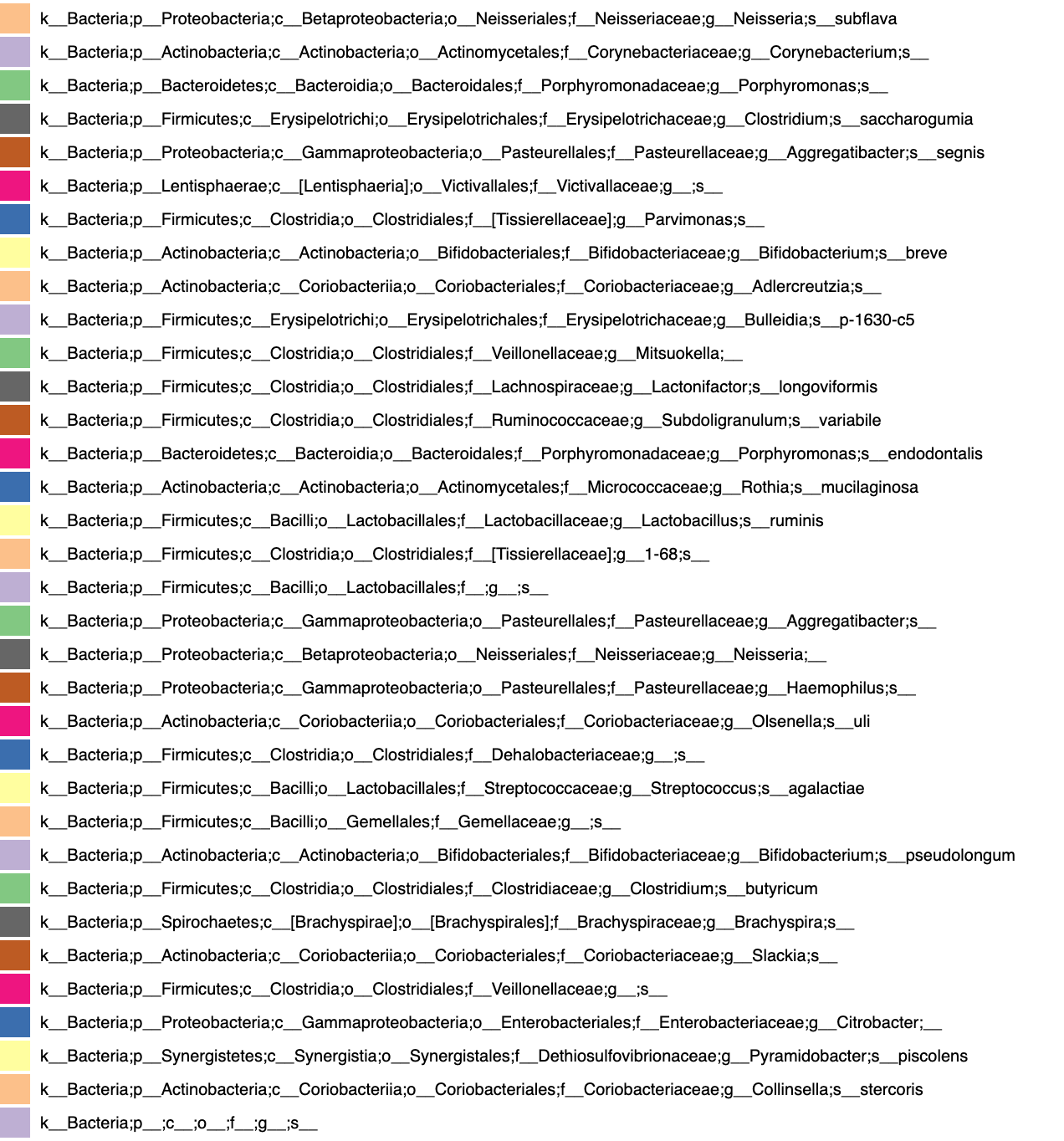


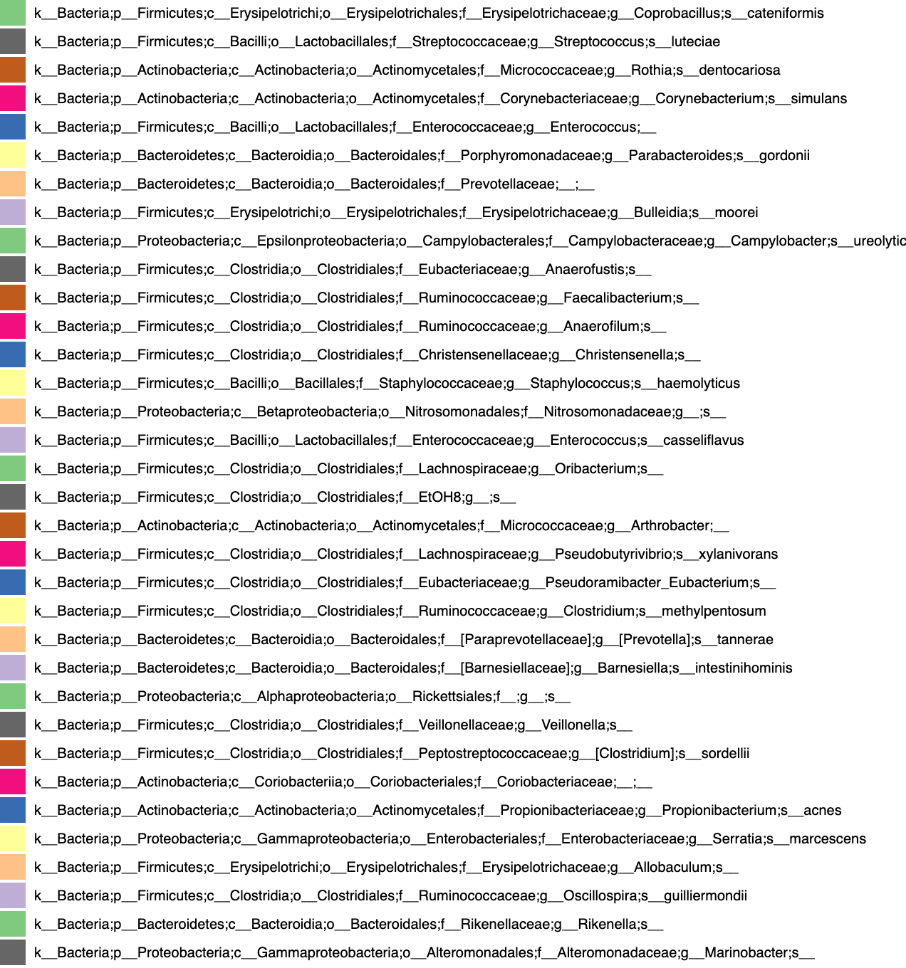


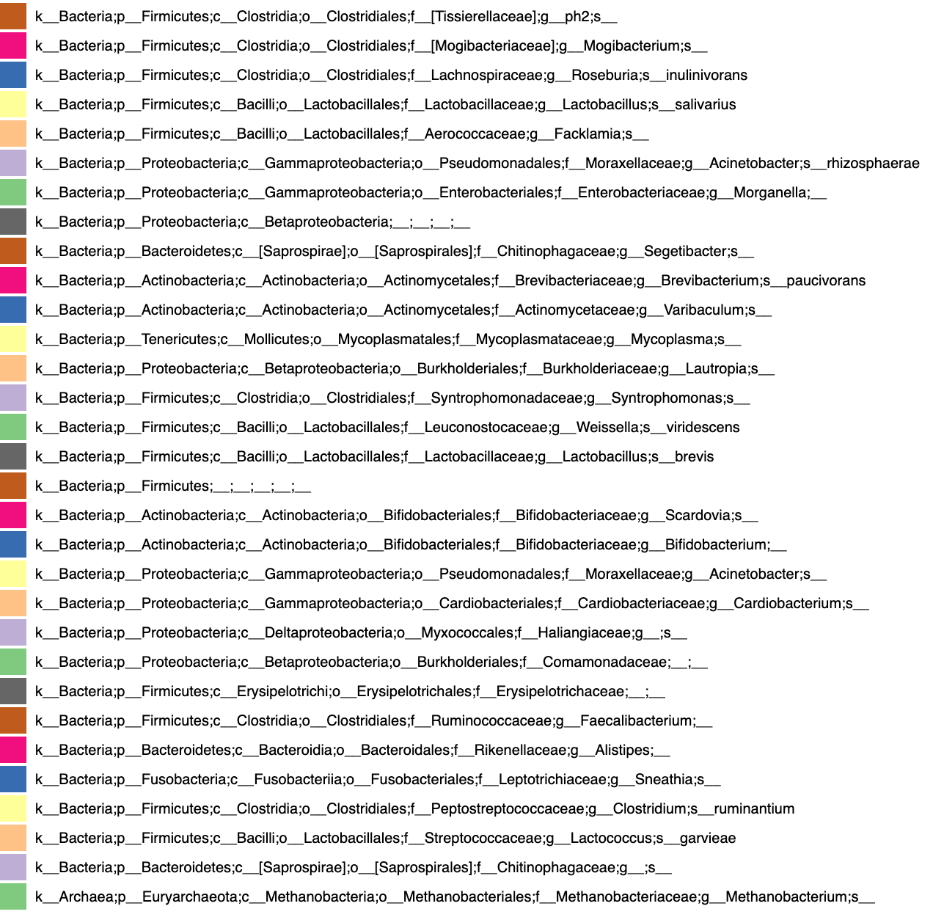


**Supplementary Figure 2. Plots of Richness (A), Shannon index (B), and Pielou index (C) with fecal calprotectin class (<250 or ≥250 µg/g) indication.**

**
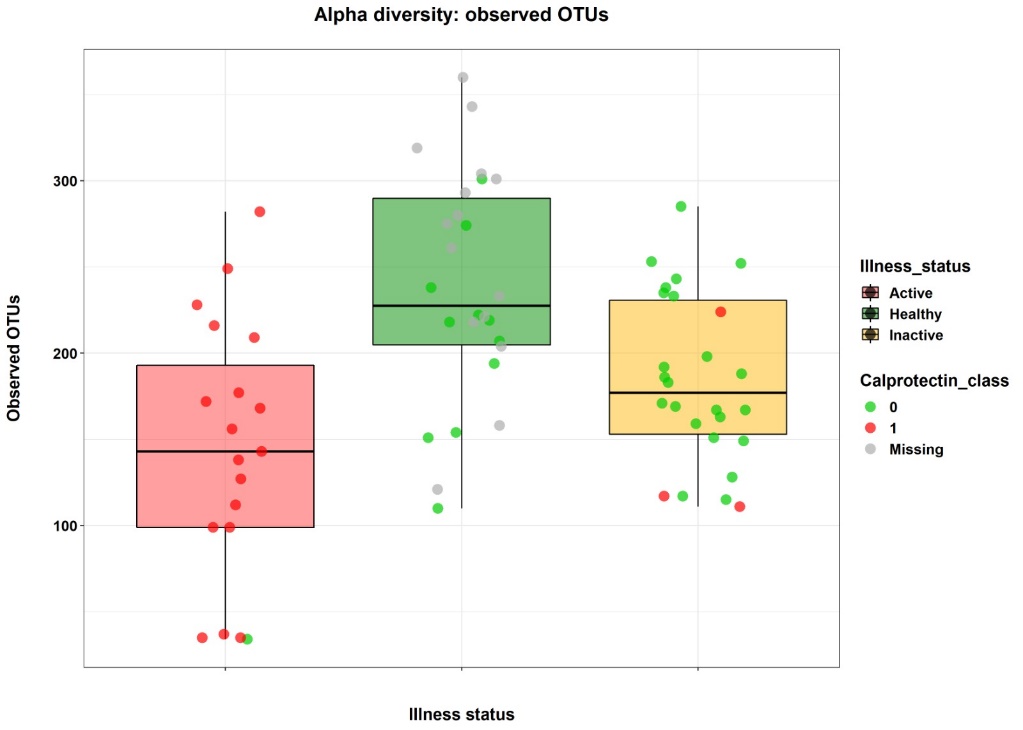
**

**B.**

**
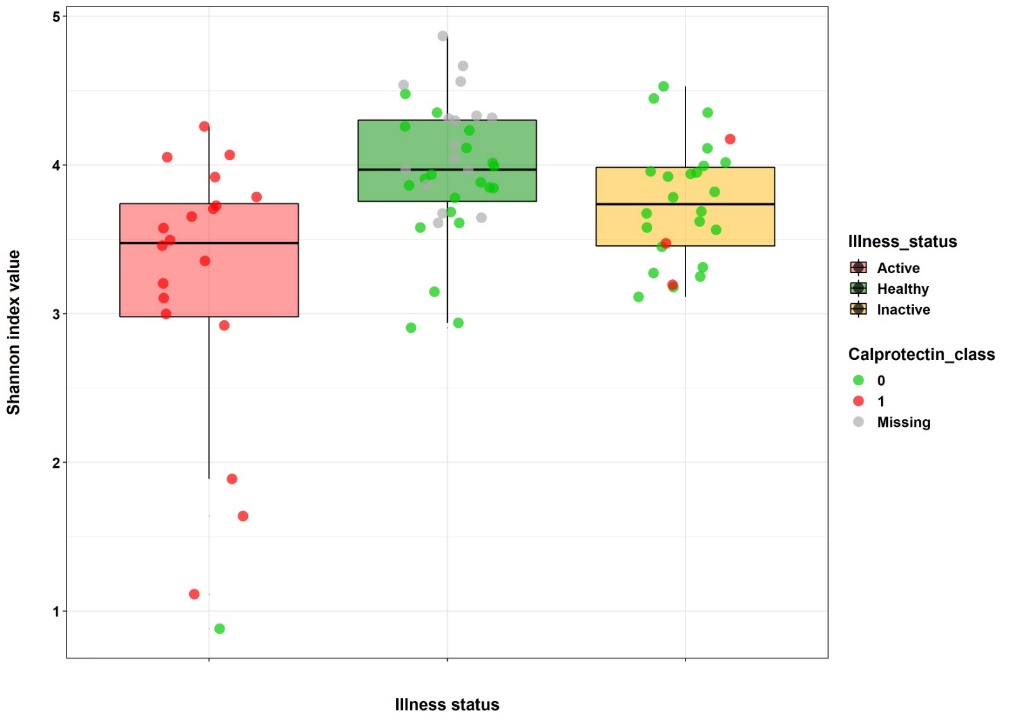
**

**C.**

**
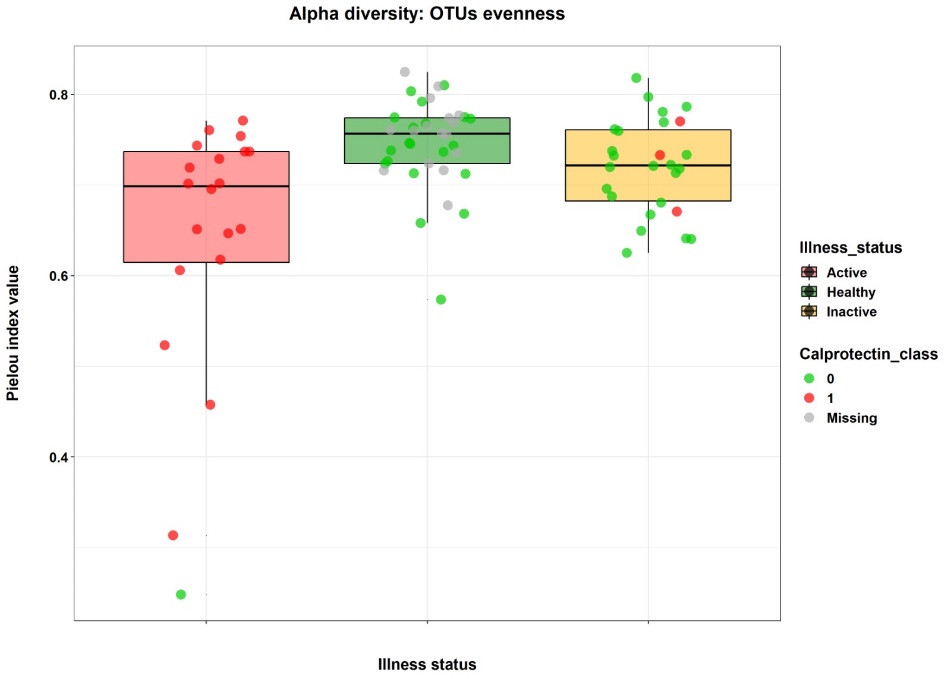
**

Points were colored corresponding to fecal calprotectin values: < 250 µg/g indicated as green and 0, ≥250 µg/g indicated as red and 1. Thus, we combined each metric quantitative information with the fecal calprotectin value. Note, some fecal calprotectin samples were missing in the healthy control group.

**Supplementary Figure 3. Dendrogram representing hierarchical clustering results obtained by using Ward algorithm on Canberra distance.**


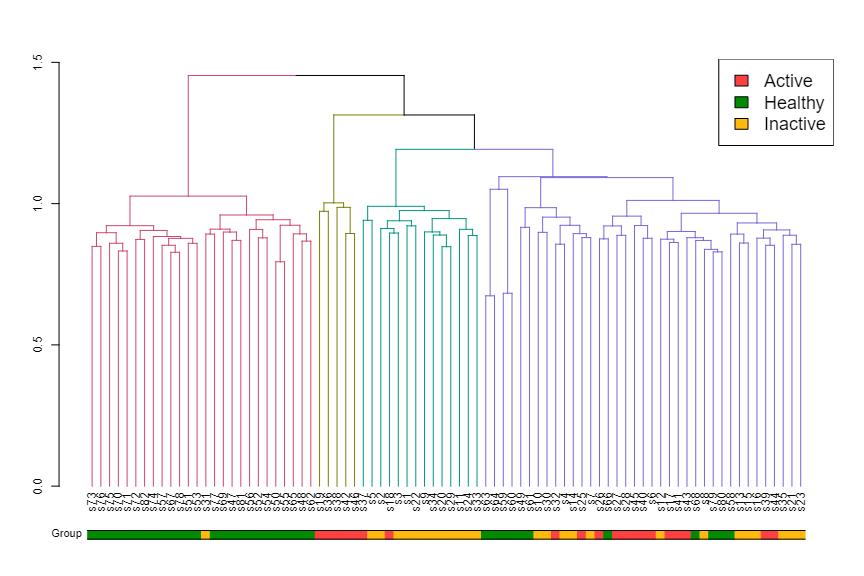


Healthy control samples are labelled in green, active UC in red and inactive UC in yellow. Blue squares highlight the four clusters obtained at cutting level equal to 1.15.
